# A deep learning-based approach toward differentiating scalp psoriasis and seborrheic dermatitis from dermoscopic images

**DOI:** 10.3389/fmed.2022.965423

**Published:** 2022-11-03

**Authors:** Zhang Yu, Shen Kaizhi, Han Jianwen, Yu Guanyu, Wang Yonggang

**Affiliations:** Inner Mongolia Medical University, Hohhot, China

**Keywords:** deep learning, scalp psoriasis, seborrheic dermatitis, dermoscopy, artificial intelligence (AI)

## Abstract

**Objectives:**

This study aims to develop a new diagnostic method for discriminating scalp psoriasis and seborrheic dermatitis based on a deep learning (DL) model, which uses the dermatoscopic image as input and achieved higher accuracy than dermatologists trained with dermoscopy.

**Methods:**

A total of 1,358 pictures (obtained from 617 patients) with pathological and diagnostic confirmed skin diseases (508 psoriases, 850 seborrheic dermatitides) were randomly allocated into the training, validation, and testing datasets (1,088/134/136) in this study. A DL model concerning dermatoscopic images was established using the transfer learning technique and trained for diagnosing two diseases.

**Results:**

The developed DL model exhibits good sensitivity, specificity, and Area Under Curve (AUC) (96.1, 88.2, and 0.922%, respectively), it outperformed all dermatologists in the diagnosis of scalp psoriasis and seborrheic dermatitis when compared to five dermatologists with various levels of experience. Furthermore, non-proficient doctors with the assistance of the DL model can achieve comparable diagnostic performance to dermatologists proficient in dermoscopy. One dermatology graduate student and two general practitioners significantly improved their diagnostic performance, where their AUC values increased from 0.600, 0.537, and 0.575 to 0.849, 0.778, and 0.788, respectively, and their diagnosis consistency was also improved as the kappa values went from 0.191, 0.071, and 0.143 to 0.679, 0.550, and 0.568, respectively. DL enjoys favorable computational efficiency and requires few computational resources, making it easy to deploy in hospitals.

**Conclusions:**

The developed DL model has favorable performance in discriminating two skin diseases and can improve the diagnosis, clinical decision-making, and treatment of dermatologists in primary hospitals.

## Introduction

As a common and recurring chronic inflammatory skin disease, psoriasis has been an affliction of over 125 million people worldwide (1). It manifests high incidence, easy recurrence, and long course, which makes a significant impact on the physical and mental health of patients. In China, more than six million diagnoses of psoriasis have been reported, and the prevalence is increasing year by year, which poses a challenge to public health. The problem is exacerbated by insufficient and unevenly distributed medical resources. It is known that the diagnosis of psoriasis relies on doctors' skills and auxiliary measures. However, most experienced dermatologists and medical devices are deployed in big cities, whereas dermatologists and general practitioners in remote areas exhibit low diagnostic accuracy for skin disease. Consequently, many patients are misdiagnosed or under delayed treatment. In this context, we aim to develop an automatic diagnosis technology for addressing the above problem.

**Figure 1:**
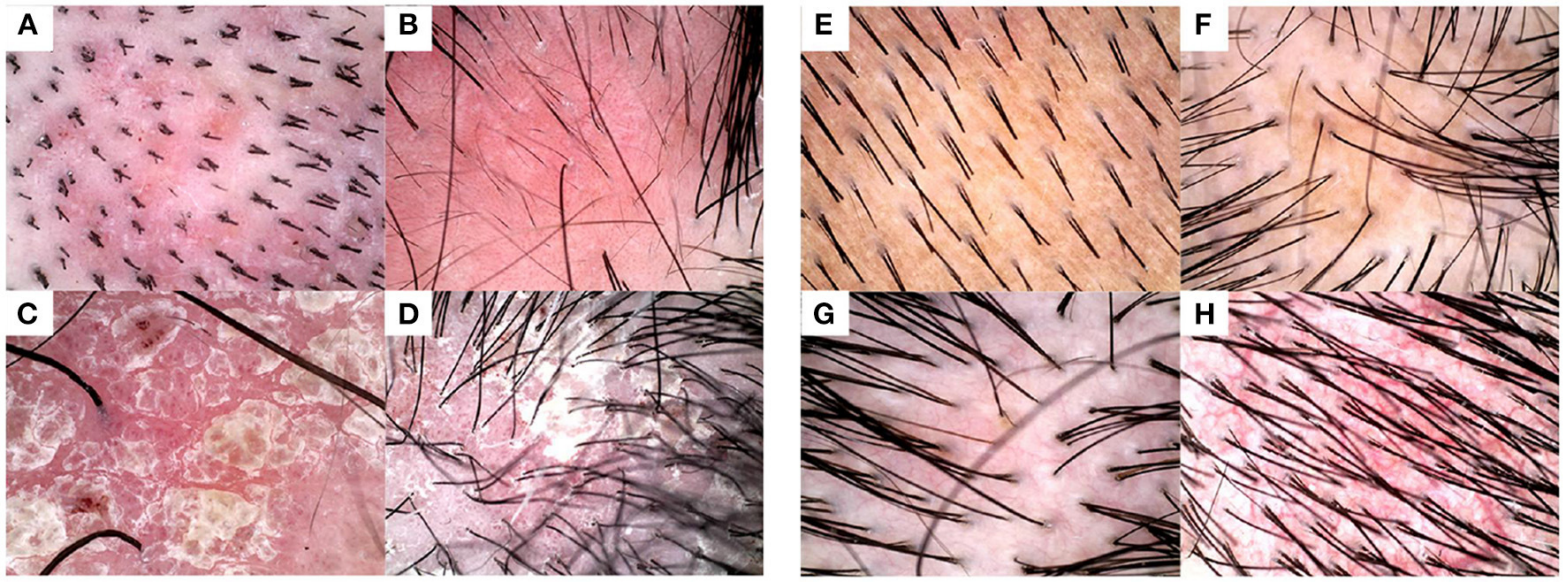
Examples of typical dermoscopic images. Typical images from **(A–D)** psoriasis lesions, wherein annular and hairpin blood vessels that have high specificity for the diagnosis can be seen under dermoscopy, and **(E–H)** seborrheic dermatitis lesions, wherein branching and atypical vessels show distinct unstructured white areas and honeycombed pigmentation networks.

An appropriate auxiliary diagnostic measure should be determined as the input of the automatic diagnosis technology. Histopathology is not regarded as the primary examination in many hospitals due to the requirement for traumatic samples and skin pathology experts, and RCM (reflectance confocal microscopy) is also unfavorable in practice owing to its high costs (2). The dermoscopy instrument, as a low-cost and noninvasive device, takes the enlarged view of lesions with simple backgrounds, few interference factors, and obvious structure, and therefore enjoys high accuracy and specificity in psoriasis lesions diagnosis. Thus, dermoscopy is adopted in this study. The characteristics of typical scalp psoriasis and seborrheic dermatitis under dermoscopy are shown in Figure 1.

Based on dermoscopic images, we develop a deep learning (DL)-based classification model for the diagnosis of skin diseases. The DL technique eliminates tedious preprocessing and complicated intermediate design by achieving data-driven end-to-end classification, which greatly simplifies method design. The representative DL technique, namely convolutional neural networks (CNNs) exhibit remarkable capability in feature extraction and thus has been widely employed for image classification (3–5). Over the past few years, a variety of DL-based medical applications have been reported, such as the assisted diagnosis of digestive tract tumors with pathology, MRI, and ultrasound images (6–9), the diagnosis of dysphagia with photographs of the anterior neck (10), and the precise location and identification of cells in microscopic images (11). In the dermatology field, DL uses trunk or longitudinal skin stratification images of patients' lesions to diagnose skin tumors, atopic dermatitis, psoriasis, and fungal infections (12–15). However, rare studies focus on scalp skin disease, since it is challenging to be accurately diagnosed due to hair interference, unobvious vascular features, and coverage of thick white scales. In particular, scalp psoriasis is one of the most affected conditions since it is hard to distinguish from seborrheic dermatitis. Motived by this, we propose a DL-based classification model for psoriasis and seborrheic dermatitis on the scalp in this paper to give diagnostic suggestions in a variety of complex situations.

The contributions of this study are summarized as follow, First, we propose a new dataset including various scalp psoriasis and scalp seborrheic dermatitis samples to train the DL model. Then, the well-trained DL model is compared with those of 5 Chinese certified dermatologists proficient in diagnosing using dermoscopy with respect to diagnostic accuracy, and we validate the DL model can benefit non-proficient doctors by improving their performance. Finally, we evaluate the feasibility of the model by assessing its computational load. Overall, the developed DL model can be used as a pre-screening tool to help the general practitioners make recommendations when faced with scalp psoriasis and scalp seborrheic dermatitis, assist dermatologists to prioritize their diagnoses, and lay the foundation for precision medicine and telemedicine consultation.

## Materials and methods

### Design and settings

**Figure 2:**
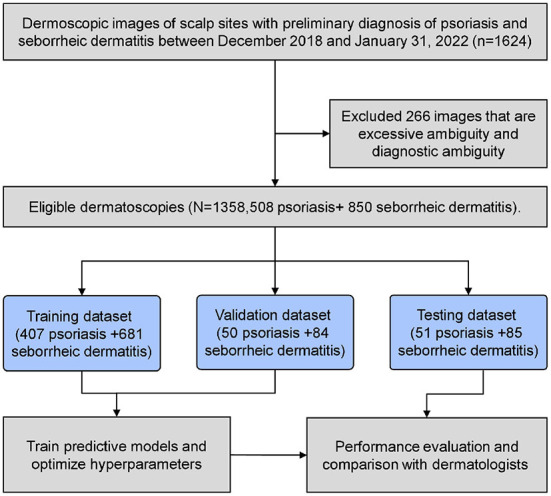
Knowledge representation tree for decision making.

In this diagnostic study, all the dermoscopic images were obtained from the Department of Dermatology, Affiliated Hospital of Inner Mongolia Medical University. The trial was reviewed and approved by the institutional ethics committee. From December 2019 to January 31, 2022, we selected a total of 1,624 dermoscopic images (obtained from 735 patients) of scalp parts preliminarily diagnosed with psoriasis and seborrheic dermatitis. The model of dermoscopy is BN-PFMF-8001, the magnification is 20–220x, and the imaging resolution is 2,592 × 1,944 pixel. During the dermoscopic image acquisition, we selected different magniations of the 20–40x interval according to the patient's lesion size. In order to enhance the generalization ability of the developed DL model, image samples for training were processed by data augmentation methods. The images were rotated randomly at [−90, 90] degrees and scaled randomly at [1,2]. These image rotations and flips were performed in MATLAB using the deep learning toolbox. And the dataset was also standardized into a Gaussian distribution. Two senior dermatologists interpreted the images. Two sixty six images were removed due to ambiguous diagnosis. They combined the pathological results and diagnostic treatment effects, and re-diagnosed and classified the dermoscopic images. When there are different opinions between the two dermatologists, another dermatologist with 7 years of experience in dermatoscopy is introduced to make a decision. The experience of 3 dermatologists in reading the test set ranged from 5 to 7 years. A total of 1,358 pictures (obtained from 617 patients) with clinically proven scalp psoriasis (508 cases) and seborrhea dermatitis (850 cases) were randomly assigned to training, validation, and test datasets (1,088/134/136 lesions). 650 of the 1,358 photos (from 331 patients) had been confirmed by pathological biopsy. The above described is shown in the workflow chart in Figure 2.

We developed a DL model based on the above 1,358 dermoscopic images of scalp sites, wherein the training and validation datasets were used for the model training, and the testing dataset was employed for the model evaluation. Owing to the powerful information extraction performance of the convolution-based deep learning model, in our methodology, image titling or subsetting operations were not involved. An image interpolation method was adopted to remap the original dermatoscopy image to the specified input size (224 × 224 pixel). To evaluate the developed DL model, we compare its diagnostic accuracy against dermatologists with various dermatoscopy experience levels, namely five dermatologists, a dermatology graduate student, and two general practitioners. Five dermatologists and one dermatology graduate student were from two hospitals affiliated with Inner Mongolia Medical University, and two general practitioners were from township hospitals in Inner Mongolia Province. Among the five dermatologists, three (60%) were male and two (40%) were female. One dermatology graduate student and two general practitioners were female. Additionally, two dermatologists have <3 years of experience in dermatoscopy, two between 3 and 5 years and one more than 5 years.

### Development of the DL diagnosis model

**Figure 3:**
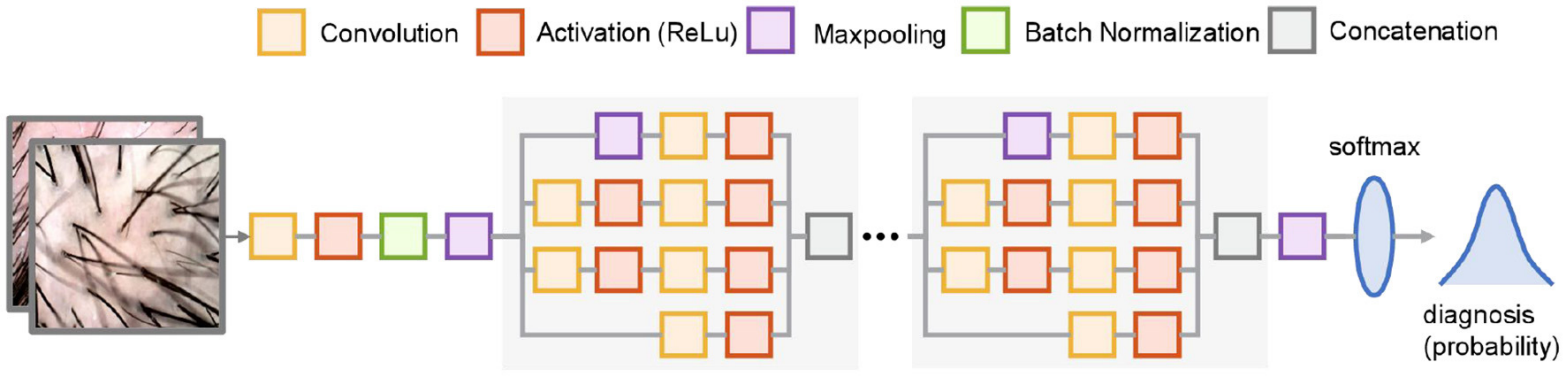
Schematic diagram of the architecture of the developed DL model. The gray boxed parts are repeated nine times and partially omitted for brevity. The model receives 224 × 224 pixel images and outputs the probability of two diseases.

The developed DL model was established based on the transfer learning technique. We employed the far-reaching deep neural network, namely GoogLeNet as the backbone of the developed model. GoogLeNet is a 22-layer deep convolutional network that is pre-trained using the ImageNet dataset for image classification. To establish the DL model, we tweaked the last convolutional layer of the GoogleNet and used the transfer learning technique to retrain the parameters of the deep network for achieving the diagnosis of psoriasis and seborrheic dermatitis. The schematic diagram of the model is shown in Figure 3. As for the learning configuration, we adopted an Adam optimizer with an initial learning rate of 1e-4 and the mean square error (MSE) loss function with L2 regularization. The training dataset was for model learning and the validation dataset was to eliminate the overfitting problem in the training process. The model training was performed on a PC with a GPU of Nvidia RTX 3070, and the scripts were written using MATLAB. More training details can be found in Supplementary materials.

### Statistical analysis

Analysis of the data was completed using SPSS (Version 25.0; Chicago, IL), with a two-sided *P*-value of <0.05 being considered statistically significant. The performance of the diagnosis of the developed model was assessed by the receiver operating characteristic (ROC) curve analysis and the area under curve (AUC). *Z* test was used to compare the differences of AUCs for different predictive results.

## Results

### Patient characteristics

A total of 1,358 patients were enrolled in the training, validation, and testing cohort. In the training (psoriasis accounted for 30.0% [407/1,088]), validation (psoriasis accounted for 37.3% [50/134]) and test (psoriasis accounted for 37.5% [51/136]) data sets, there was no significant difference in the prevalence of scalp psoriasis and seborrheic dermatitis (χ^2^= 2.933, *P* = 0.231). There were 508 patients with psoriasis, aged 35.4 ± 11.2. Men account for 265/508 and women for 243/508. There were 850 patients with seborrheic dermatitis, aged 32.6 ± 9.9. Men account for 476/850 and women account for 374/850.

### Diagnostic performance comparison between the DL model and dermatologists

**Figure 4:**
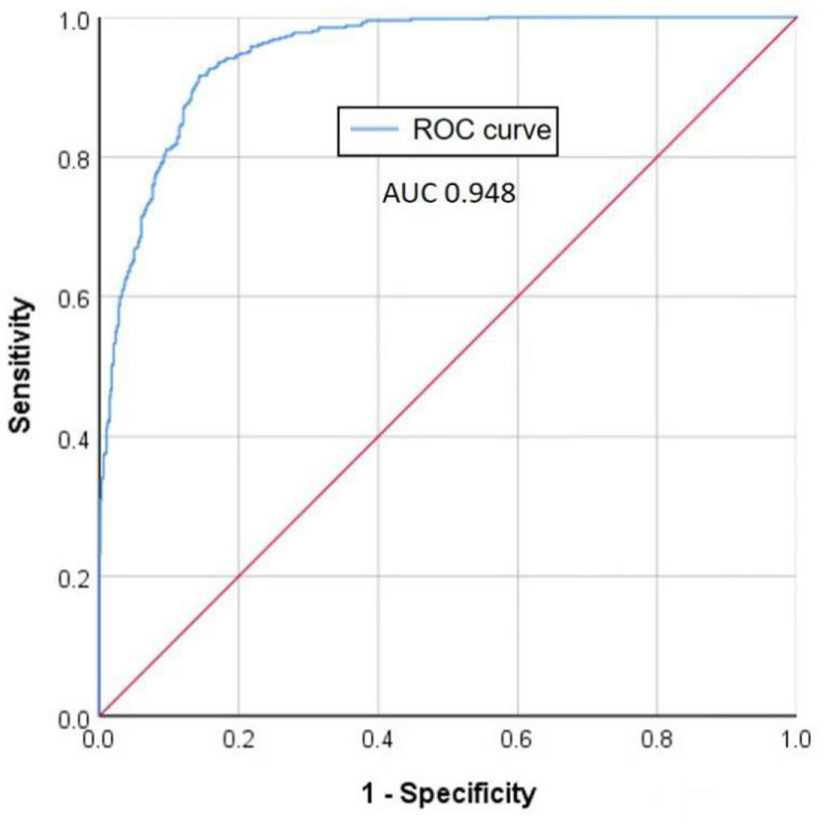
ROC curve of the training set of the developed DL model.

To evaluate the training performance of the developed DL model, the ROC curve performed using the training dataset is illustrated in Figure 4, where a 0.948 AUC indicates our model achieves a favorable accuracy.

**Figure 5:**
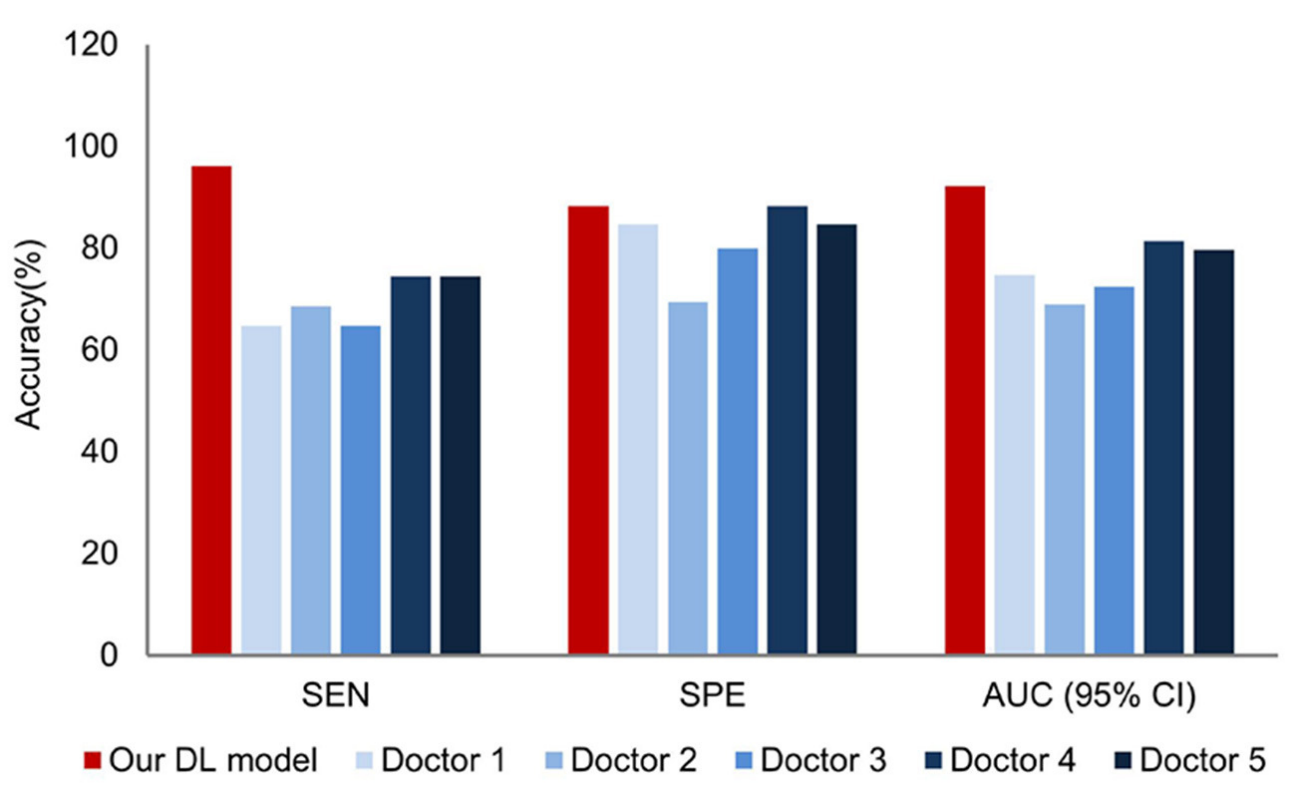
Comparison of the DL model and dermatologist in the testing dataset.

**Figure 6:**
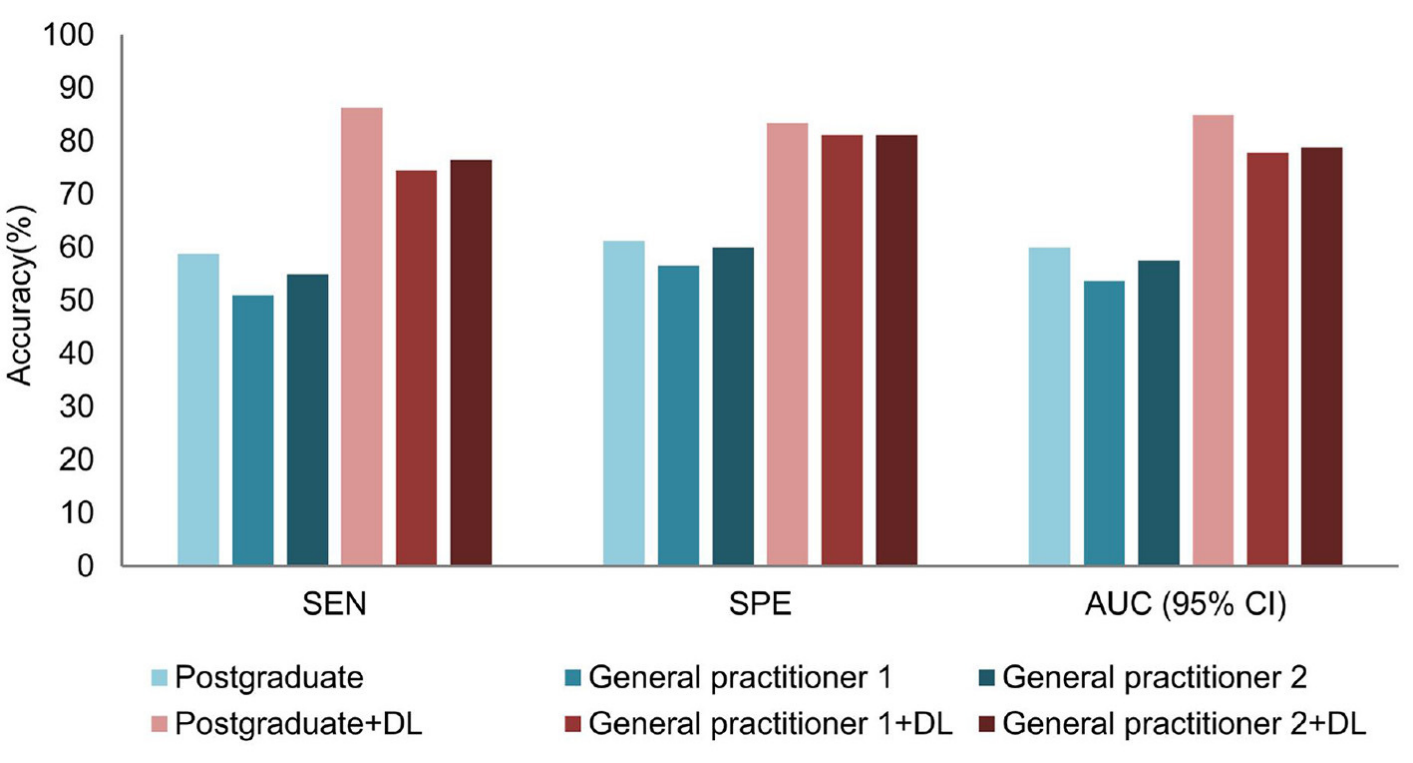
Comparison before and after DL model assistance for graduate students and general practitioners in the test dataset.

In the test data set, when comparing the diagnostic performance of the prediction model and dermatologists with different years of experience in the diagnosis of seborrheic dermatitis and psoriasis on the scalp, the results showed that the diagnostic ability of the DL model was better than those of five dermatologists. Figure 5 summarizes the sensitivity, specificity and AUC of the combined model and dermatologist. As shown in Figure 5, the AUC values reflect the level of diagnostic accuracy. As a result of limited medical resources in practice, it is common for general practitioners and dermatology students to diagnose and treat skin conditions, and their performance in dermoscopy and dermatology diagnosis is inferior to dermatologists. Thus, it is necessary to investigate the effect of the proposed DL model on the diagnostic performance of general practitioners and dermatology students. As shown in Figure 6, the dermatology graduate student and two general practitioners were able to improve the accuracy of dermatoscopy identification with the assistance of the DL model. The AUCs of the dermatology graduate student, general practitioner 1, and general practitioner 2 increased from 0.600, 0.537, 0.575 to 0.849, 0.778, 0.788, respectively. Assisted by the DL model, their diagnostic ability was even comparable to that of dermatologists.

**Figure 7:**
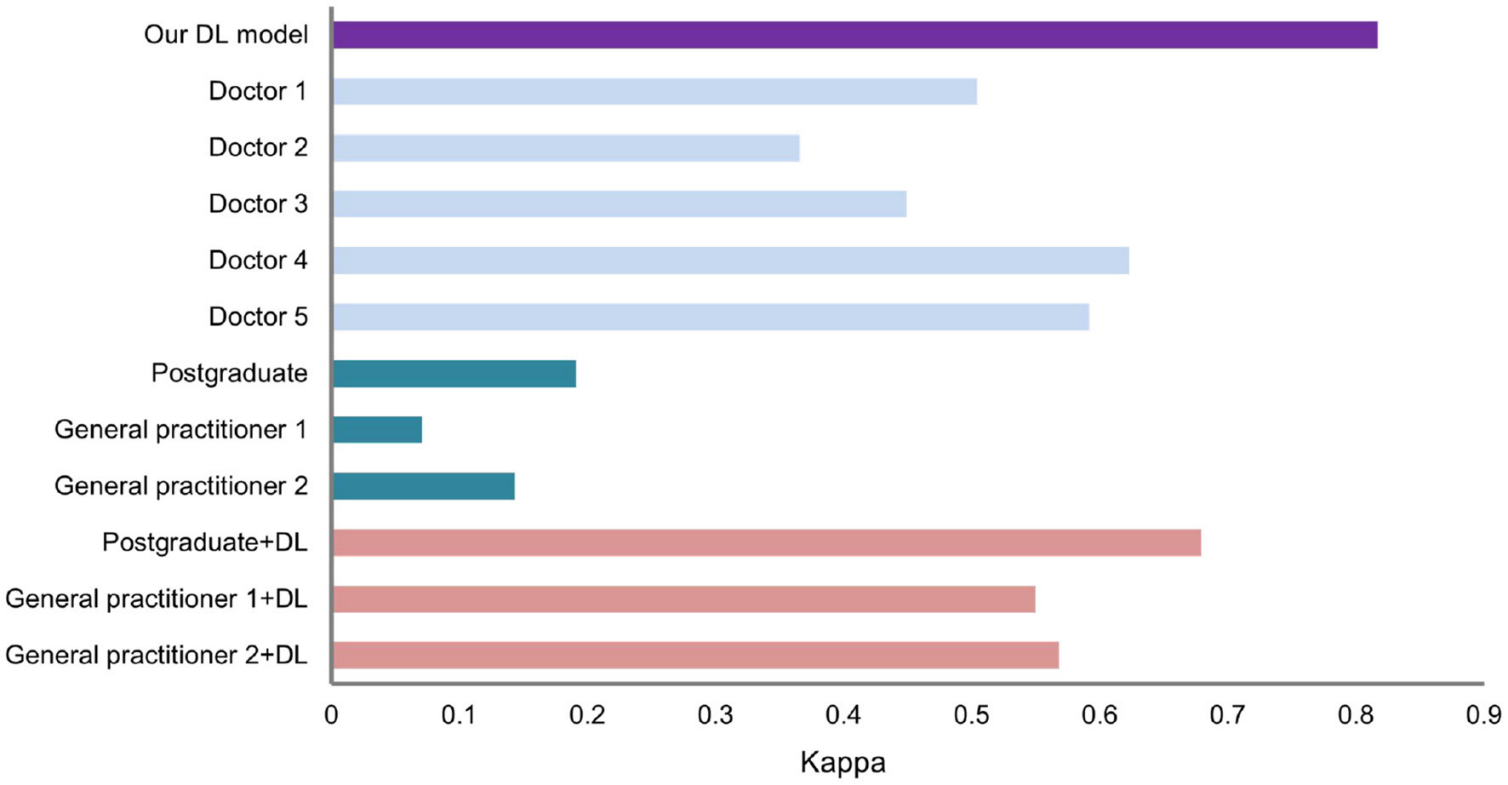
Kappa value of the DL model and dermatologist.

Furthermore, the consistency of the dermatology graduate student and two general practitioners significantly improved as shown in Figure 7. In the absence of the DL help, their kappa values were 0.191, 0.071, and 0.143, respectively. When assisted by the DL model, kappa values were raised to 0.679, 0.550, and 0.568, respectively, which were equivalent to dermatologists who have studied dermatoscopy.

**Figure 8:**
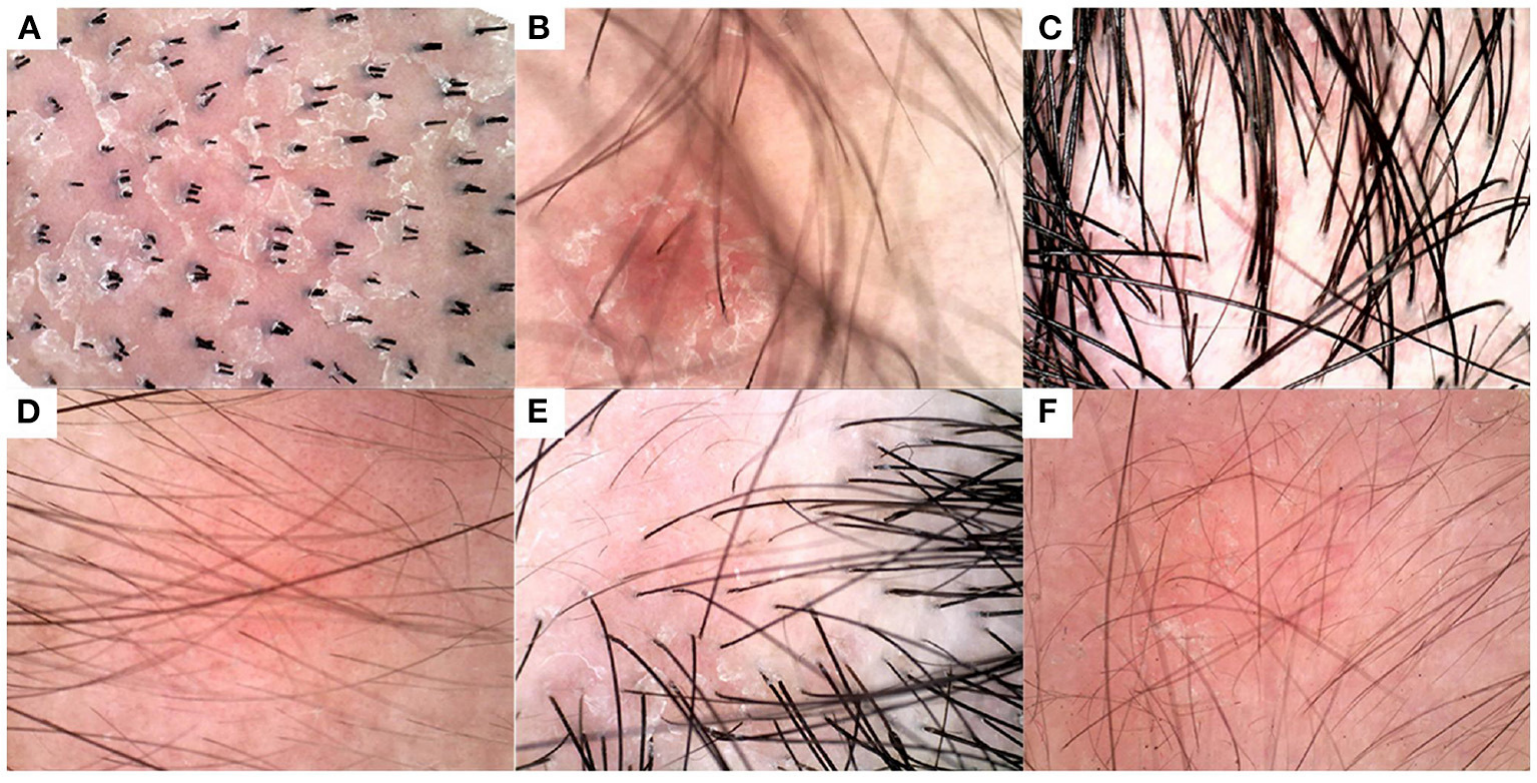
Diagnosis results of DL model and humans (five physicians) on some indistinguishable lesion images. The below figures are represented in ground truth disease type and correctly diagnosis rate of humans: **(A)** seborrheic dermatitis, 40%, **(B)** seborrheic dermatitis, 60%, **(C)** seborrheic dermatitis, 0%, **(D)** psoriasis, 20%, **(E)** psoriasis, 40%, and **(F)** psoriasis, 40%. For comparison, the DL model correctly diagnosed all six images.

Furthermore, the developed DL model can achieve accurate diagnosis regarding lesion images with few typical features. Six lesion images with vague diagnoses are demonstrated in Figure 8, where no obvious signs of typical psoriasis or seborrheic dermatitis were found, such as round vessels, hairpin vessels, branch vessels, or atypical vessels. For these images, the probabilities of correct diagnosis by five dermatologists were 40, 60, 0, 20, 40, and 40%, respectively, whereas the DL model achieved the correct diagnosis in all lesions. It is probably because DL can eliminate dandruff, hair, and other influencing factors based on other information in lesions.

**Figure 9:**
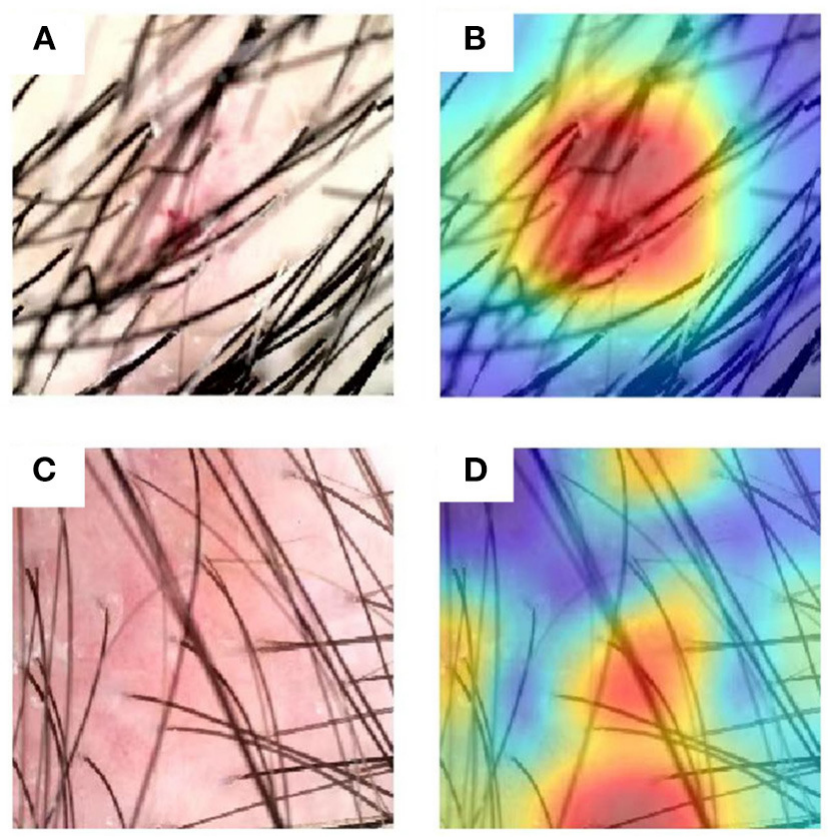
Exemplary images that were misdiagnosed and corresponding attention distributions of the DL model. **(A,B)** An example image of seborrheic dermatitis and the associated attention distribution from the DL model, respectively. **(C,D)** An example image of psoriasis and the associated attention distribution from the DL model respectively.

Nonetheless, it is significant to analyze the failure cases of the DL model. As shown in Figure 9, the weights of the last intermediate layer of the DL model were extracted and plotted to visualize the attention of the model, where a red area indicates higher attention whereas a blue area indicates lower attention. Figures 9B,D show that the DL model paid the most attention to the blood scab and non-vascular area, respectively. However, in a human expert's opinion, vascular characteristics should be the preferred diagnosis mark. To some extent, these attention distributions explain the DL model's misdiagnosis. Comparing the correctly diagnosed and incorrectly diagnosed images in Figures 8, 9, it is indicated that a better cleanup of the affected scalp area could potentially improve the DL model's accuracy.

Overall, the diagnostic accuracy of the DL model is still higher than that of 5 dermatologists trained in dermoscopy with respect to dermoscopic images with less obvious typical characteristics.

### Computational performance evaluation of the DL model

The computation performance of the developed DL model is of great importance to evaluate its applicability in practice. The DL model has higher computational efficiency on a GPU in general, but it can also be deployed on CPU-only devices when considering the hospital setting. A GPU of Nvidia RTX 3070 and a CPU of Intel i3 8100, respectively served as the computing device of the DL model to simulate the real-world deployment environment. Then we calculated the clock time consumption of two devices in completing the diagnosis and provided the results in Table 1. Our results suggest that the GPU is indeed faster at executing models. However, the computational cost of single dermoscopic images in the CPU environment is also very low. In this way, we validated that the developed DL model exhibits favorable real-time computational efficiency and can be employed in real-world hospitals for diagnosis.

**Table 1:** Clock time consumption of the DL model.

**Inputs**	**Device**	**Mean (s)**	**Standard deviation (s)**
One sample (1 image)	GPU	0.0246	0.0061
Total test set (136 images)	GPU	0.5501	0.0150
One sample (1 image)	CPU	0.0488	0.0014
Total test set (136 images)	CPU	2.2209	0.2380

Means and standard deviations are obtained based on 30 runs.

## Discussion

Accurate identification of psoriasis and seborrheic dermatitis on the scalp is of great significance to numerous patients in China. An accurate and rapid examination will greatly reduce patients' anxiety by cutting out biopsy and pathological confirmations and avoiding incorrect treatment. In this study, we developed a deep learning model to diagnose scalp psoriasis and seborrheic dermatitis under dermoscopy. The DL model exhibits better diagnostic performance than most dermatologists. In the diagnosis of psoriasis on scalp sites, DL models have higher sensitivity and specificity compared with dermatologists, thus meeting the clinical requirements for accurate diagnosis. Furthermore, the DL model can benefit dermatologists with varying levels of training and experience: one dermatology graduate student and general practitioners can greatly improve their diagnosis accuracy by using the DL model.

The results show the superiority of our DL model in the diagnostic accuracy of scalp psoriasis and seborrheic dermatitis, which could potentially benefit dermatologists. Based on the diagnosis results of 51 pictures of psoriasis cases in the test set, the model reached 96.1% sensitivity. Therefore, the DL model can prevent the delay of patients' treatment, tackle the development of the disease course, and improve the prognosis when encountering patients with ambiguous diagnoses. In addition, we can see from Figures 5, 6 that the DL model not only outperformed the diagnostic accuracy of the five dermatologists, but also assists graduate students and general practitioners to significantly improve the diagnostic accuracy.

Despite the resource consumption of the training process, the well-trained model can easily be implemented in simple digital skin examination systems, such as personal computers. The developed model can identify the basic characteristics of the two diseases, eliminate hair interference, and enable remote diagnosis. The global pandemic of COVID-19 has strained medical resources and made it hard for patients to access hospitals (especially those that offer higher-level treatments). In this context, increasing numbers of dermatological patients seek online advice due to the visibility of most skin diseases. However, for online treatment, patients must provide images of their lesions. The image quality of the scalp surface and the difficulty of removing scales complicate medical judgment. The DL model developed in this paper process 224 × 224 pixel images and can achieve accurate and rapid diagnosis with low image resolution.

## Limitations

This study selected dermoscopic images from the dermatology department of the dermatology hospital of the Inner Mongolia Medical University. It is not excluded that the results of this study may not fully represent all manifestations of scalp psoriasis and seborrheic dermatitis, and there may be the problem of generalization. By collecting images from the same well-trained dermatologist, we eliminate the variance caused by the proficiency of different dermatologists in the operation of dermatoscopy. Nevertheless, it should be considered to reduce the requirements for photo quality and the photography process, so that the algorithm can be more easily applied to practical situations. In addition, the diagnosis of dermatologists generally needs to start with the patient's medical history. This study focuses on visual information and does not consider the influencing factors of skin lesions such as the family history of the disease, the degree of activity, and obesity. A total of 650 of the 1,358 photos in this study (from 331 patients) have been confirmed by pathological biopsy; however, there may be diagnostic bias in the samples that have not been confirmed by pathological biopsy. Dermoscopy selection, hair coverage, and other factors can also affect diagnosis accuracy. The images we selected in the training, validation, and test sets were all from fair-skinned people, all of whom were Chinese. In the future, these factors will be integrated into the DL model to further improve diagnostic accuracy.

## Conclusions

The DL model has high specificity (88.2%), sensitivity (96.1%), and AUC (0.922) in differentiating scalp psoriasis and seborrheic dermatitis. In terms of the AUC values, the accuracy of the DL model is higher than that of dermatologists who have undergone dermoscopy training. The DL model can also help the dermatology graduate students and general practitioners to improve the diagnostic accuracy of scalp psoriasis and seborrheic dermatitis. With the assistance of the DL model, the AUCs of one dermatology graduate student and two general practitioners have been increased by 0.249, 0.241, 0.213, respectively.

## Data availability statement

The raw data supporting the conclusions of this article will be made available by the authors, without unduereservation.

## Ethics statement

The studies involving human participants were reviewed and approved by Inner Mongolia Medical University. Written informed consent for participation was not required for this study in accordance with the national legislation and the institutional requirements. Written informed consent was not obtained from the individual(s) for the publication of any potentially identifiable images or data included in this article.

## Author contributions

ZY: conceptualization, methodology, software, validation, formal analysis, writing, and funding acquisition. HJ, SK, WY, and YG: resources, data curation, and investigation. All authors contributed to the article and approved the submitted version.

## Conflict of interest

The authors declare that the research was conducted in the absence of any commercial or financial relationships that could be construed as a potential conflict of interest.

## Publisher's note

All claims expressed in this article are solely those of the authors and do not necessarily represent those of their affiliated organizations, or those of the publisher, the editors and the reviewers. Any product that may be evaluated in this article, or claim that may be made by its manufacturer, is not guaranteed or endorsed by the publisher.

## References

[1] National Psoriasis Foundation Data. Available online at: www.sporiasis.org (accessed October 2017).

[2] Lian-Sheng ZH Zhi-Ping WE Yan-Qun LI. Sensitivity and specificity of Munro microabscess detected by reflectance confocal microscopy in the diagnosis of psoriasis vulgaris. J Dermatol. (2012) 39:282–3. 10.1111/j.1346-8138.2011.01366.x 21973117

[3] He K Zhang X Ren S Sun J. Deep residual learning for image recognition. In: Proceedings of the IEEE Conference on Computer Vision and Pattern Recognition. Las Vegas, NV: IEEE (2016). 10.1109/CVPR.2016.90

[4] Krizhevsky A Sutskever I Hinton GE. Imagenet classification with deep convolutional neural networks. Adv Neural Inform Process Syst. (2012) 60:84–90. 10.1145/3065386

[5] Chandra SS Bran Lorenzana M Liu X Liu S Bollmann S Crozier S. Deep learning in magnetic resonance image reconstruction. J Med Imag Radiat Oncol. (2021) 65:564–77. 10.1111/1754-9485.13276 34254448

[6] Wang J Liu X. Medical image recognition and segmentation of pathological slices of gastric cancer based on Deeplab v3+ neural network. Comput Methods Programs Biomed. (2021) 207:106210. 10.1016/j.cmpb.2021.106210 34130088

[7] Wang P Liu X Berzin TM Brown JR Liu P Zhou C. Effect of a deep-learning computer-aided detection system on adenoma detection during colonoscopy (CADe-DB trial): a double-blind randomised study. Lancet Gastroenterol Hepatol. (2020) 5:343–51. 10.1016/S2468-1253(19)30411-X 31981517

[8] Zhang W Yin H Huang Z Zhao J Zheng H He D. Development and validation of MRI-based deep learning models for prediction of microsatellite instability in rectal cancer. Cancer Med. (2021) 10:4164–73. 10.1002/cam4.3957 33963688 PMC8209621

[9] Wang L Song H Wang M Wang H Ge R Shen Y. Utilization of ultrasonic image characteristics combined with endoscopic detection on the basis of artificial intelligence algorithm in diagnosis of early upper gastrointestinal cancer. J Healthcare Eng. (2021). 10.1155/2021/2773022 34880973 PMC8648460

[10] Sakai K Gilmour S Hoshino E Nakayama E Momosaki R Sakata N. A machine learning-based screening test for sarcopenic dysphagia using image recognition. Nutrients. (2021) 13:4009. 10.3390/nu13114009 34836264 PMC8622012

[11] Gol S Pena RN Rothschild MF Tor M Estany J. A polymorphism in the fatty acid desaturase-2 gene is associated with the arachidonic acid metabolism in pigs. Sci Rep. (2018) 8:1–9. 10.1038/s41598-018-32710-w 30254373 PMC6156218

[12] Haenssle HA Fink C Schneiderbauer R Toberer F Buhl T Blum A. Man against machine: diagnostic performance of a deep learning convolutional neural network for dermoscopic melanoma recognition in comparison to 58 dermatologists. Ann Oncol. (2018) 29:1836–42. 10.1093/annonc/mdy166 29846502

[13] Esteva A Kuprel B Novoa RA Ko J Swetter SM Blau HM. Dermatologist-level classification of skin cancer with deep neural networks. Nature. (2017) 542:115–8. 10.1038/nature21056 28117445 PMC8382232

[14] Czajkowska J Badura P Korzekwa S Płatkowska-Szczerek A. Deep learning approach to skin layers segmentation in inflammatory dermatoses. Ultrasonics. (2021) 114:106412. 10.1016/j.ultras.2021.106412 33784575

[15] Tognetti L Bonechi S Andreini P Bianchini M Scarselli F Cevenini G. A new deep learning approach integrated with clinical data for the dermoscopic differentiation of early melanomas from atypical nevi. J Dermatol Sci. (2021) 101:115–22. 10.1016/j.jdermsci.2020.11.009 33358096

