# Supplementary material for: A deep learning-based approach toward differentiating scalp psoriasis and seborrheic dermatitis from dermoscopic images
Source: Front Med (Lausanne). 2022 Nov 3;9:965423. doi: 10.3389/fmed.2022.965423 (PMC9669613; doi:10.3389/fmed.2022.965423)
Supplement: Supplementary file 1 [file Data_Sheet_1.docx]

**Supplementary Materials**

*A. Training process of the DL model*

The training dataset was processed with data augmentation techniques for better DL performance. Specifically, training images were randomly rotated in the range of [-90, 90] degrees, and the sizes of images were randomly rescaled. The data distributions of the two classes are shown in Fig. S1, where the sample ratios are roughly the same across training and validation sets. Other hyper-parameters for training the DL model are provided in Table S1.


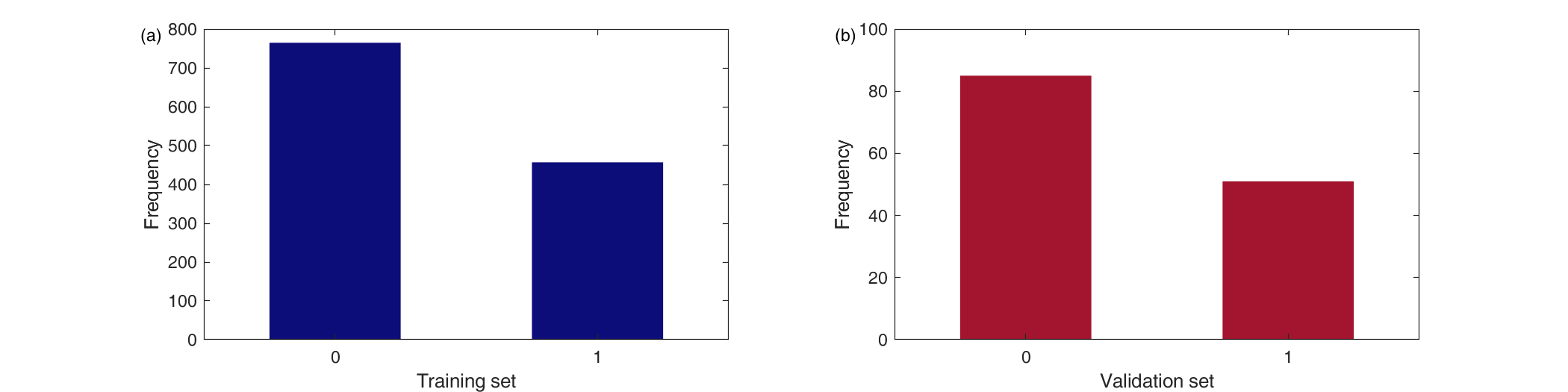


Fig. S1. Distribution of the amount of data, where classes 0 and 1 represent psoriasis and seborrheic dermatitis, respectively. (a) Training set. (b) Validation set.

**Table S1. Hyperparameters for training the DL model**

| Parameter | Value |
| --- | --- |
| Minibatch Size | 32 |
| Optimizer | Adam |
| Loss function | Mean Squared Error Loss |
| Initial Learning Rate | 1e-4 |
| L2 Regularization | 1e-4 |
| Gradient Decay Factor | 0.9 |

The training process is depicted in Fig. S2. According to the ascending curve in subfigure (a), the developed DL model can steadily improve its diagnosis accuracy on the training set. More importantly, a successful training process is indicated by the synchronized increases in accuracy for the validation set. The training convergence is indicated by the loss values in subfigure (b). The asymptotic diagnosis accuracy of both sets is around 90%, demonstrating the competence of the developed DL model.


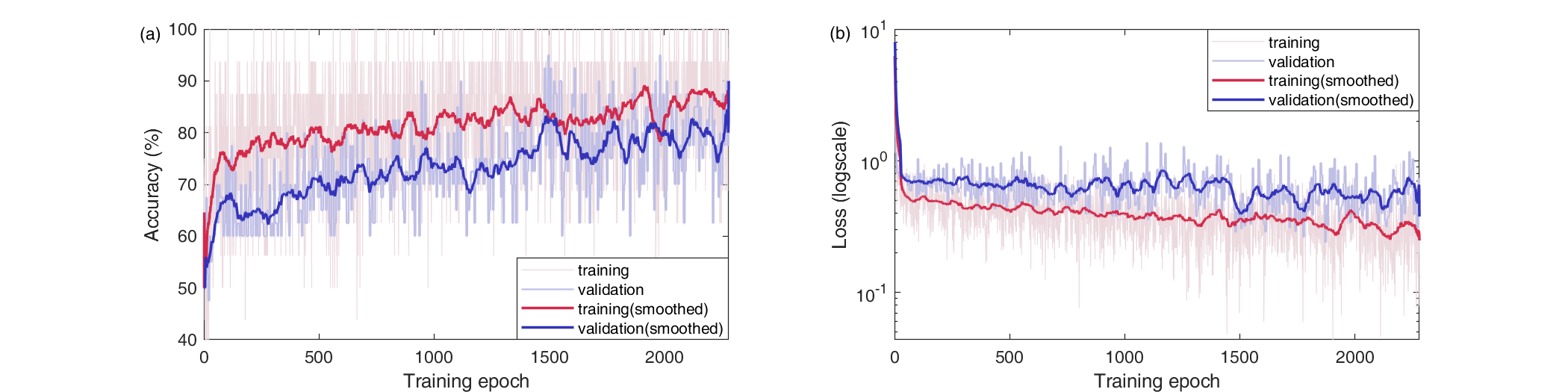
Fig. S2. Training process of the DL model. (a) Diagnosis accuracy with respect to the training process. (b) Loss value of the DL model with respect to the training process.

1. *Statistical chart of the calculated results of the statistical section*

**Table1a. Comparison of the DL model and dermatologist in the testing dataset**

|  | SEN | SPE | PPV | NPV | AUC *(95% CI)* | *P* value |
| --- | --- | --- | --- | --- | --- | --- |
| Our DL model | 96.1 (49/51) | 88.2 (75/85) | 83.1 (49/59) | 97.4 (75/77) | 0.922 (0.871-0.972) | NA |
| Doctor 1 | 64.7 (33/51) | 84.7 (72/85) | 71.7 (33/46) | 80.0 (72/90) | 0.747 (0.657-0.837) | 0.0005 |
| Doctor 2 | 68.6 (35/51) | 69.4 (59/85) | 57.4 (35/61) | 78.7 (59/75) | 0.690 (0.597-0.783) | <0.0001 |
| Doctor 3 | 64.7 (33/51) | 80.0 (68/85) | 66.0 (33/50) | 79.1 (68/86) | 0.724 (0.632-0.815) | 0.0001 |
| Doctor 4 | 74.5 (38/51) | 88.2 (75/85) | 79.2 (38/48) | 85.2 (75/88) | 0.814 (0.733-0.895) | 0.0131 |
| Doctor 5 | 74.5 (38/51) | 84.7 (72/85) | 74.5 (38/51) | 84.7 (72/85) | 0.796 (0.713-0.879) | 0.0054 |
| Postgraduate | 58.8 (30/51) | 61.2 (52/85) | 47.6 (30/63) | 71.2 (52/73) | 0.600 (0.501-0.699) | <0.0001 |
| General practitioner 1 | 51.0 (26/51) | 56.5 (48/85) | 41.3 (26/63) | 65.8 (48/73) | 0.537 (0.437-0.638) | <0.0001 |
| General practitioner 2 | 54.9 (28/51) | 60.0 (51/85) | 45.2 (28/62) | 68.9 (51/74) | 0.575 (0.475-0.674) | <0.0001 |
| Postgraduate+DL | 86.3 (44/51) | 83.5 (71/85) | 75.9 (44/58) | 91.0 (71/78) | 0.849 (0.778-0.920) | 0.0501 |
| General practitioner 1+DL | 74.5 (38/51) | 81.2 (69/85) | 70.4 (38/54) | 84.1 (69/82) | 0.778 (0.694-0.863) | 0.0021 |
| General practitioner 2+DL | 76.5 (39/51) | 81.2 (69/85) | 70.9 (39/55) | 85.2 (69/81) | 0.788 (0.705-0.871) | 0.0033 |

Data for sensitivities, specificities, PPVs, and NPVs were percentages, with numerators and denominators in parentheses.

SEN sensitivity, SPE specificity, AUC area under the receiver operating characteristic curve, PPV positive predictive value, NPV negative predictive value, CI confidence interval

*P* value: Compared with Predictions

**Table 1b. Kappa value of the DL model and dermatologist**

|  | Kappa | *P* value |
| --- | --- | --- |
| Our DL model | 0.817 | <0.001 |
| Doctor 1 | 0.504 | <0.001 |
| Doctor 2 | 0.366 | <0.001 |
| Doctor 3 | 0.449 | <0.001 |
| Doctor 4 | 0.623 | <0.001 |
| Doctor 5 | 0.592 | <0.001 |
| Postgraduate | 0.191 | 0.024 |
| General practitioner 1 | 0.071 | 0.399 |
| General practitioner 2 | 0.143 | 0.091 |
| Postgraduate+DL | 0.679 | <0.001 |
| General practitioner 1+DL | 0.550 | <0.001 |
| General practitioner 2+DL | 0.568 | <0.001 |
